# High-Resolution Adaptive Optics Transscleral Flood Illumination Imaging Allows Phenotyping of Age-Related Macular Degeneration †

**DOI:** 10.3390/cells14171308

**Published:** 2025-08-24

**Authors:** Safa Mohanna, Leila S. Eppenberger, Oliver Pfäffli, Sohrab Ferdowsi, Sonja Simon-Zoula, Christoph Amstutz, Lucas M. Bachmann, Michael A. Thiel, Martin K. Schmid

**Affiliations:** 1 Eye Clinic, Cantonal Hospital of Winterthur, 8400 Winterthur, Switzerland; 2 Eye Clinic, University Hospital of Bern, 3010 Bern, Switzerland; 3 Augenarztpraxis Cham, 6330 Cham, Switzerland; 4 EarlySight S.A., 1202 Geneva, Switzerland; 5 Eye Clinic, Cantonal Hospital of Lucerne, 6000 Lucerne, Switzerland; 6 Medignition A.G., 8004 Zurich, Switzerland; 7 Epidemiology, Biostatistics and Prevention Institute Zurich, University of Zurich, 8001 Zurich, Switzerland; 8 Faculty of Health Sciences and Medicine, University of Lucerne, 6005 Lucerne, Switzerland

**Keywords:** age-related macular degeneration (AMD), adaptive optics, transscleral flood illumination, retinal imaging, optical coherence tomography, AMD phenotyping, geographic atrophy, retinal pigment epithelium

## Abstract

Adaptive optics transscleral flood illumination (AO-TFI) enables in vivo imaging of the retinal pigment epithelium (RPE) at near-cellular resolution. In this study, we evaluated its potential as a phenotyping tool in age-related macular degeneration (AMD) by analyzing disease-associated structural patterns and their correlation with optical coherence tomography (OCT) features. We examined AO-TFI images from 120 eyes diagnosed with either early-to-advanced dry AMD (including geographic atrophy, GA) or neovascular AMD (nvAMD). Images were graded by a masked reader, and patterns were matched to corresponding OCT findings. Four consistent morphologic patterns were identified: atrophy, pre-atrophy, soft drusen, and reticular pseudodrusen. Morphometric quantification of hyporeflective regions showed progressive changes in perimeter, diameter, and area from reticular pseudodrusen to soft drusen and pre-atrophy, returning to lower values in atrophy. Distinct nvAMD-specific signatures were not identified. AO-TFI offers a practical, high-resolution complement to OCT, AO-SLO, and AO-OCT for phenotypic characterization of AMD in clinical settings.

## 1. Introduction

Multimodal imaging has become essential in the diagnosis and management of age-related macular degeneration (AMD), particularly using optical coherence tomography (OCT), fundus autofluorescence, and fluorescein angiography. These modalities enable structural characterization of disease progression and the treatment response, especially in advanced disease. However, their ability to resolve fine, cellular-level changes in the outer retina and retinal pigment epithelium (RPE) remains limited. Subtle pathological alterations—such as early RPE disruption or photoreceptor misalignment—may remain undetected until irreversible damage has occurred. This lack of high-resolution, layer-specific biomarkers presents a key obstacle to early detection, precise phenotyping, and timely intervention in AMD [1,2].

In recent years, adaptive optics (AO) technologies have provided a means to visualize the retina at near-cellular resolution in vivo. Among these, AO scanning laser ophthalmoscopy (AO-SLO) and AO optical coherence tomography (AO-OCT) have demonstrated impressive capabilities to resolve microscopic details, including cone photoreceptors, RPE mosaics, and drusen morphology [3]. AO-SLO, in particular, has been instrumental in mapping the photoreceptor mosaic and in studying dynamic structural changes over time [4]. Split-detector AO-SLO can visualize cones’ inner segments even when outer segments are lost, making it especially useful in diseases involving outer retinal disruption [4,5]. However, its small field of view (typically 1–2°) and sensitivity to eye motion require image averaging and complex registration, which can limit its practicality in routine clinical settings [4,5,6].

Similarly, AO-OCT offers the advantage of depth-resolved visualization, with high axial resolution (~3–5 µm) capable of distinguishing laminar changes in the photoreceptors, RPE, and choroid [6]. It has proven particularly valuable in assessing outer retinal integrity, especially when combined with AO-SLO [7]. Nevertheless, acquisition time, sensitivity to motion artifacts, and the need for complex image processing pipelines currently limit its broader clinical implementation. Moreover, both AO-SLO and AO-OCT are primarily research tools in most centers, with limited integration into standard diagnostic workflows.

In contrast, adaptive optics transscleral flood illumination (AO-TFI) has emerged as a novel, complementary modality that aims to combine cellular resolution with practical, wide-field clinical imaging. Introduced by Laforest et al. in 2020, AO-TFI employs tangential near-infrared illumination through the sclera and captures scattered light through the pupil using a flood illumination camera system [8,9]. The resulting images exhibit high-contrast reflectance profiles that enhance structural features—particularly those related to the RPE and subretinal space—across a larger field of view (6.7° × 6.7°) with short acquisition times (<10 s). While AO-TFI does not offer depth resolution and lacks true phase imaging, its practical advantages lie in its robustness to motion, simplicity of acquisition, and integration with standard ophthalmic exam workflows [3,8,9].

AO-TFI has demonstrated its ability to visualize photoreceptors and RPE mosaics in healthy eyes, but its clinical utility in retinal disease—particularly AMD—remains under active investigation [8,9]. The RPE plays a central role in AMD pathophysiology, and early subclinical changes such as RPE thinning, dropout, or morphological irregularity may precede overt atrophy or fluid accumulation. Capturing these early alterations may enhance our ability to define pre-atrophic stages, understand lesion progression, and differentiate overlapping phenotypes. Similarly, distinguishing drusen subtypes—such as soft drusen, cuticular drusen, and reticular pseudodrusen—has been shown to carry prognostic value and reflect divergent disease pathways [10,11]. However, conventional imaging struggles to resolve these deposits in sufficient detail for reliable subtyping.

## 2. Materials and Methods

We conducted a single-center prospective observational study at the Eye Clinic of the Cantonal Hospital, Lucerne, Switzerland. The study protocol (ClinicalTrials.gov: NCT04912622; kofam.ch: SNCTP000004502) was approved by the Regional Ethics Committee (EKNZ2020-02454) and adhered to the tenets of the Declaration of Helsinki, ISO 14155, and national regulatory standards.

A convenience sample of patients with clinical diagnoses of age-related macular degeneration (AMD), including early, intermediate, and advanced stages, both geographic atrophy (GA) and neovascular AMD (nvAMD), was recruited. Patients were classified based on the Beckman classification system using multimodal imaging, including OCT and color fundus photography [12]. Classification was performed by trained retina specialists at the time of inclusion. Exclusion criteria included history of epilepsy or inability to comply with the examination protocol (e.g., due to cognitive or neurologic impairments). Written informed consent was obtained from all participants.

While no formal power calculation was performed at the study initiation, the inclusion of 120 eyes was based on logistical feasibility and patient availability. Post hoc analysis indicated that this sample size was adequate to detect medium effect sizes in pattern distribution analyses with sufficient statistical power.

All participants underwent standard ophthalmological assessment, including slit-lamp biomicroscopy, OCT (Spectralis; Heidelberg Engineering, Heidelberg, Germany), fundus autofluorescence (AF; Spectralis Blue Peak module, Heidelberg, Germany), and optical biometry (OA-2000; Tomey, Nagoya, Japan), prior to AO-TFI imaging.

Refractive error, pupil size, lens status, and media clarity were recorded at the time of AO-TFI imaging. All eyes achieved at least a 6 mm pupil diameter following pharmacologic dilation to ensure sufficient illumination. Media clarity was assessed clinically using slit-lamp biomicroscopy. Only eyes with clear ocular media or mild lens changes (e.g., early cataract without posterior subcapsular or dense nuclear opacities) and no vitreous haze were included to preserve image quality. Refractive error was recorded as a spherical equivalent, with a mean of −0.75 D (±1.9 D; range −5.00 to +2.50). Lens status was available for 80 eyes: 62.5% were pseudophakic and 37.5% phakic. These values are summarized in Table 1.

AO-TFI images were acquired using the Cellularis AO retinal camera (version 2.0, EarlySight SA, Geneva, Switzerland). This system uses two near-infrared light-emitting diodes (λ = 850 nm), positioned nasally and temporally on the sclera, to deliver transscleral oblique illumination. Light scattered from retinal and subretinal structures is collected through the pupil using a flood-illumination optical path. Images obtained from each illumination angle are summed to increase contrast and reduce directional artifacts. This technique does not involve split-detection or phase-gradient reconstruction. The resulting images offer dark-field-like contrast that enhances structural features. Additional contrast optimization is performed through software-based post-processing integrated into the imaging pipeline. This system can acquire a 6.7° × 6.7° field of view in under 10 s.

Image focus was adjusted based on the subject’s spherical equivalent refraction, with additional manual correction to optimize clarity. Five predefined macular zones were imaged per eye, guided by an internal fixation target (580 nm LED). For participants with low visual acuity, an external fixation target and a wide-field camera mode were used to assist with alignment and region registration.

Image quality was assessed based on absence of motion artifacts and overall clarity. Images were included if they had no motion blur across the central 80% of the field. No particular signal-to-noise ratio was utilized. A total of 12 images (10%) were reacquired due to insufficient quality.

AO-TFI images were analyzed by a single trained grader masked to all OCT findings and AMD diagnoses. Grading was performed using Fiji (ImageJ, version 2.5.0; U.S. National Institutes of Health) [13]. Patterns were identified and categorized based on their reflectance profiles (hypo- or hyperreflective), spatial distribution, and morphologic features. Four distinct patterns emerged: blobs, spots, clumps, and drops. Each was correlated with specific OCT findings in a bidirectional fashion.

For each macular zone, AO-TFI patterns were registered to corresponding OCT *en face* projections using vascular landmarks and retinal orientation. Correlation was performed both from AO-TFI to OCT and vice versa using Fiji and HEYEX 2 (Heidelberg Engineering, Heidelberg, Germany) [12]. *En face* overlays and multimodal alignment were used to verify correspondence across modalities.

Proprietary software developed by EarlySight SA was utilized for automatic image quality assessment as well as morphometric analyses. The quality-assessment feature of the software uses Machine Learning (ML) to indicate the presence of blur, noise, out-of-focus areas, imaging artefacts, or retinal vessels that would occlude the analysis. This provides a numerical measure for quality for all image pixels, and areas below a certain threshold are considered to be of low quality and hence excluded from the analysis.

Similarly, the morphometric analysis functionality of the software uses Machine Learning to identify and segment salient retinal entities, such as hyporeflective or hyperreflective areas. The segmented areas are then morphometrically analyzed with common shape features such as area, perimeter, circularity, solidity, etc.

The Machine Learning pipelines used within the proprietary software follow standard best practices within this field, e.g., common splitting ratios between train, validation and test sets; ensuring low train-validation gaps during training to avoid overfitting; using typical loss functions such as cross-entropy or MSE, where appropriate; using typical optimizers and deep learning frameworks, etc.

To further validate the performance of the automatic quantification pipeline, completely independently from the Machine Learning pipeline described above, five medical students were trained by an AO-TFI expert to independently count retinal cells within 90 image crops (of size 300 by 300). The automatic ML-based grading was found to be always providing counting values between the 5 independent human graders. No data from the graders were used as labels for the ML algorithm, as this was a completely independent pipeline. The mean percentage difference among the five human graders was 19.4%.

To compare the morphometric values of the discussed patterns in this paper, we used the Mann–Whitney U test, as well as the T-test with standard values from the Scipy stats library within the Python ecosystem (scipy.stats.mannwhitneyu and scipy.stats.ttest_ind functions, Version 3.12). The returned *p*-values of these tests, i.e., to compare a pair of AO-TFI patterns with values from a morphometric descriptor, were compared with a standard threshold of *p* = 0.01 to assess statistical significance.

## 3. Results

A total of 120 eyes from 70 participants (59% women; mean age: 78 years, SD of 8) were analyzed (Table 1). Based on multimodal imaging, 62 eyes (52%) were classified as early-to-advanced dry AMD (including geographic atrophy, GA) and 58 eyes (48%) as neovascular AMD (nvAMD). Importantly, as is well-established, nvAMD and GA are not mutually exclusive and can coexist in late-stage AMD. Thus, the classification was used to ensure a structured clinical overview, though phenotypic overlap was expected.

Across the cohort, four recurring AO-TFI patterns were identified: blobs, spots, clumps, and drops. These patterns frequently appeared in combination, with multiple types present in a single eye in some cases. Only the most consistently identifiable and clinically relevant patterns are reported here.

**Figure 1:**
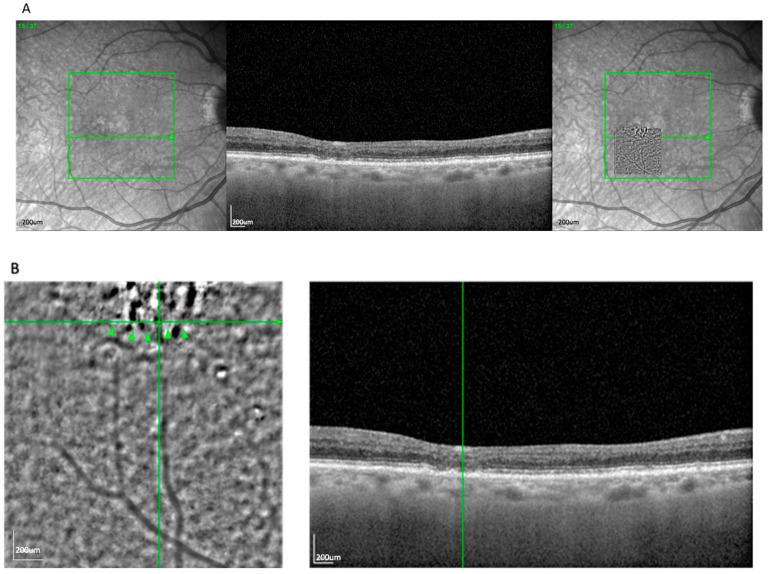

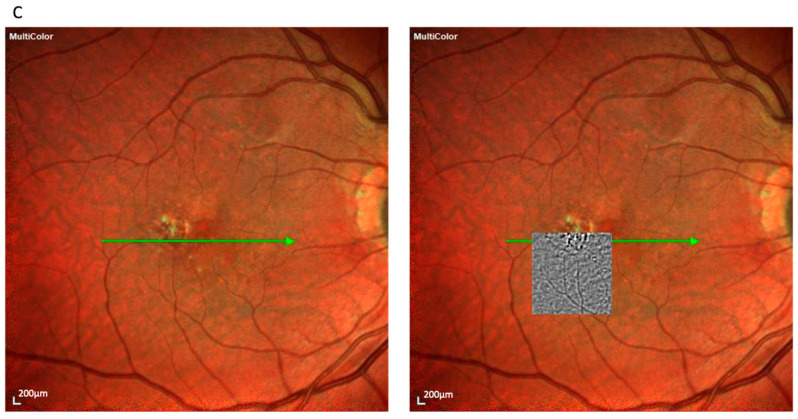
(**A**) Correlation of a blob lesion visible on an AO-TFI image in PR mode with the OCT finding using vessel structure for orientation. (**B**) Visualization of the blob and high-magnification view of the correlated atrophy zone. (**C**) Overlay of the blob with a multicolor fundus image to a zone of atrophy, using vessel structure for orientation.

The most frequent AO-TFI pattern was the blob, observed in 63 eyes (52.5%). It was characterized by a well-delineated circular zone of heterogeneous reflectivity, consistent with an extracellular deposit located between or within the RPE layers. Figure 1A demonstrates the blob lesion and its correlation with underlying OCT changes, often indicating areas of complete RPE and photoreceptor loss [14]. In the OCT, these lesions correspond to zones of established atrophy. *En face* overlays using vascular landmarks and multimodal imaging confirmed this alignment. The AO-TFI pattern corresponding to atrophy (blob) closely matched areas classified as cRORA based on OCT criteria defined by the CAM consensus [15,16]. The “blob” pattern was nearly evenly distributed between AMD subtypes (50.8% GA, 49.2% nvAMD), reflecting the known overlap between atrophic and neovascular disease in late-stage AMD.

**Figure 2:**
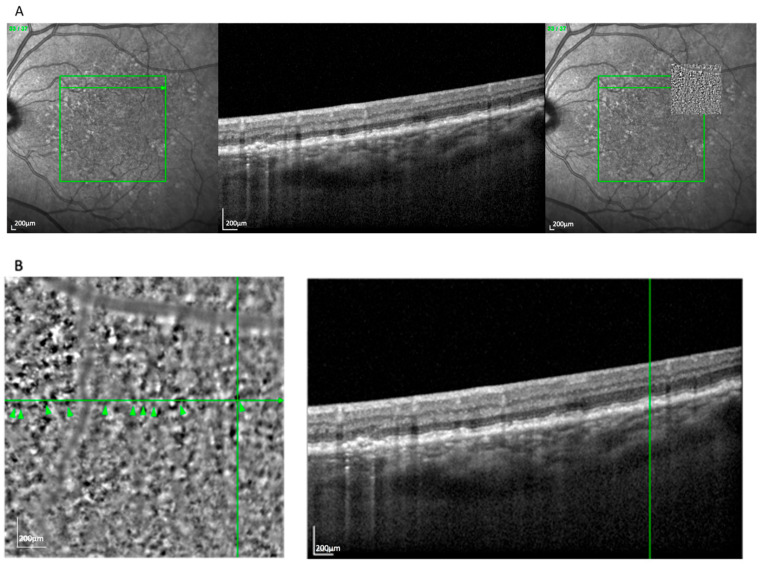
(**A**) Correlation of spot lesions visible on an AO-TFI image in RPE mode with the OCT finding using vessel structure for orientation. (**B**) Visualization of spots and high-magnification view of the correlated pre-atrophy zone. The green arrowheads indicate prominent hyperreflective clusters forming the “spots” pattern.

The spot pattern appeared in 39 eyes (32.5%) and consisted of a homogeneous distribution of small, bright clusters (typically 20–50 per image) without a defined border (Figure 2). These were associated with pre-atrophic changes in the OCT, such as localized RPE disruption and early photoreceptor irregularities. Notably, this pattern was observed in both the nvAMD and dry AMD subgroups, consistent with its representation of an early degenerative phenotype. The spot pattern showed features consistent with iRORA, including RPE irregularity and early photoreceptor disruption. This pattern occurred more often in GA (69.2%) than in nvAMD (30.8%).

**Figure 3:**
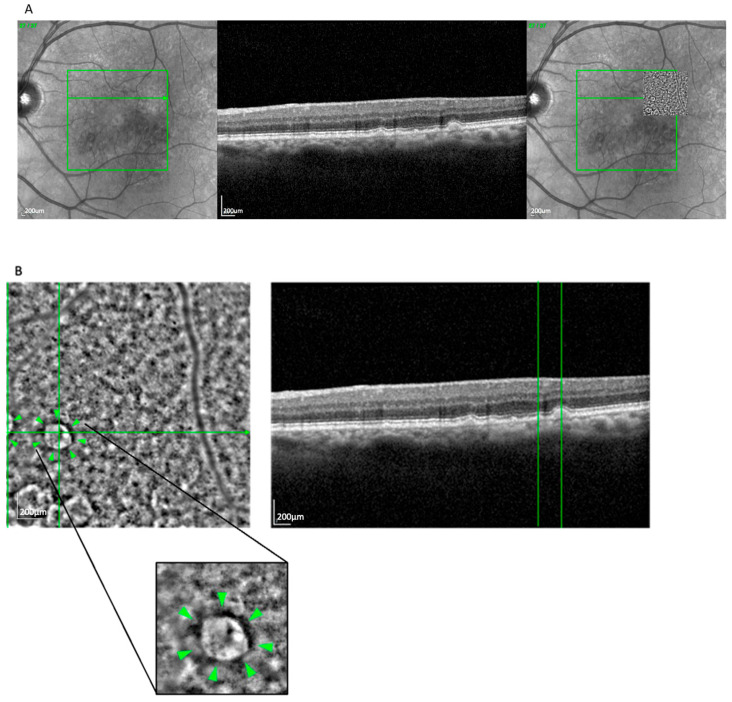
(**A**) Correlation of a clump lesion visible on an AO-TFI image in RPE mode with the OCT finding using vessel structure for orientation. (**B**) Visualization of clumps and high-magnification view of the correlated soft drusen. Green lines highlight corresponding on between en face and cross-sectional view. Arrowheads show the Pattern Clump.

The clump pattern (Figure 3) was seen in 31 eyes (25.8%) and featured a mosaic of similarly sized (~230 μm), well-organized hyperreflective clusters beneath the presumed RPE layer. This pattern corresponded with soft drusen, based on size, location, and *en face* OCT correlation. The overlying RPE signal was often elevated or irregular, consistent with drusen-induced displacement [17]. Clumps were most commonly observed in nvAMD cases (74.2%) compared to GA (25.8%).

**Figure 4:**
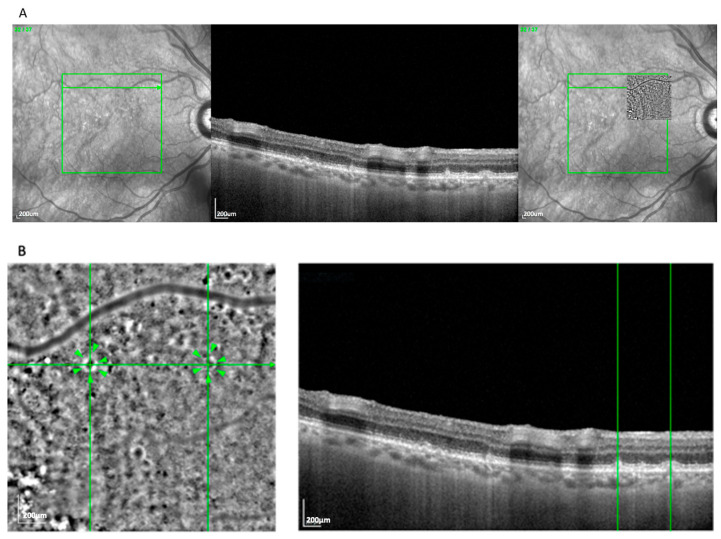
(**A**) Correlation of drop lesions visible on an AO-TFI image in RPE mode with the OCT finding using vessel structure for orientation. (**B**) Visualization of drops and high-magnification view of the correlated reticular pseudodrusen. Green lines highlight corresponding on between en face and cross-sectional view. Arrowheads show the Pattern Drops.

The drop pattern (Figure 4), observed in 14 eyes (11.6%), presented as several well-separated, homogeneously sized hyporeflective zones, often located above the RPE layer and surrounded by hyperreflective rims. This appearance aligned with reticular pseudodrusen, particularly stage 3 morphology, as seen on OCT [18]. The localization and structural profile further supported this classification. This pattern was more frequently seen in GA (71.4%) than in nvAMD (28.6%).

Note on Other Drusen Types: While soft and reticular pseudodrusen were the predominant drusen types observed, cuticular drusen were occasionally noted in individual cases. A dedicated analysis of these less-common patterns is under development for a future report.

**Figure 5:**
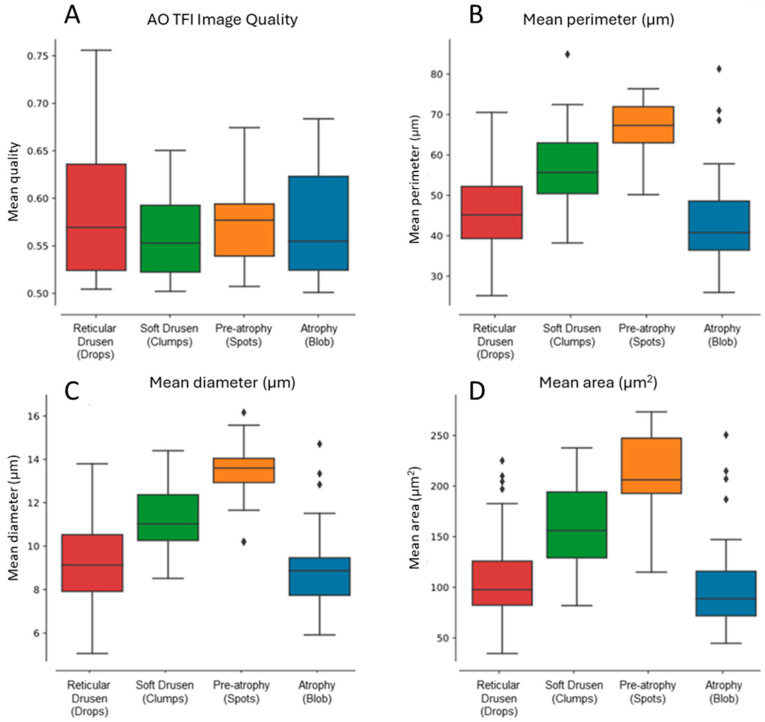
(**A**) Analysis of image quality across selected images for morphometric analysis across patterns. (**B**) Quantitative analysis of the mean perimeter, (**C**) mean diameter, and (**D**) mean area across pattern types.

Morphometric analysis of hyporeflective zones was conducted across all four patterns (Figure 5). The results demonstrated progressive increases in mean perimeter, diameter, and area measurements from reticular pseudodrusen to soft drusen and pre-atrophy. Interestingly, these values declined again in the atrophy group, approaching those seen with reticular pseudodrusen (Figure 5B–D).

This non-linear trend may reflect early accumulation of extracellular material (drusen), structural remodeling (pre-atrophy), and eventual loss of structure in complete atrophy. Results were consistent across eyes with similar image quality (Figure 5A). While these metrics (diameter, perimeter, and area) are mathematically related, each offers a slightly different representation of the spatial profile and was retained to aid interpretation.

To assess clinical relevance, pattern distribution was stratified by AMD subtype (nvAMD vs. GA). All four patterns were represented across both subtypes. However, atrophy (blob pattern) was most prevalent in eyes with GA, while spots and clumps were seen across both nvAMD and dry AMD eyes. This distribution reinforces the overlapping nature of phenotypes in late-stage AMD and supports AO-TFI’s potential for detecting shared structural signatures across disease pathways.

## 4. Discussion

In this study, we systematically evaluated adaptive optics transscleral flood illumination (AO-TFI) as a phenotyping tool in age-related macular degeneration (AMD), focusing on structural reflectance patterns that emerge from tangential illumination of retinal layers. Across a clinically diverse cohort, we identified four recurring AO-TFI patterns—atrophy (blob), pre-atrophy (spots), soft drusen (clumps), and reticular pseudodrusen (drops)—each correlated with corresponding morphological features on OCT.

Interestingly, atrophy was the most common pattern, despite the inclusion of both dry AMD and neovascular AMD (nvAMD). While this initially seemed counterintuitive, it aligns with clinical evidence that GA and nvAMD frequently co-occur in late-stage AMD [5]. Rather than viewing these as mutually exclusive entities, our findings support the idea that phenotypic overlap is common and that distinguishing them may be more useful as a conceptual framework than as a rigid classification. AO-TFI appears to capture structural hallmarks shared by both pathways, highlighting its potential value in cross-phenotype monitoring.

Another important finding was that AO-TFI enabled us to differentiate between drusen subtypes, particularly soft drusen and reticular pseudodrusen, based on spatial arrangement, reflectance pattern, and OCT correlation [11,19]. While cuticular drusen were occasionally identified, their presentation was inconsistent and less prominent in this dataset. For clarity and reproducibility, we limited our primary classification to the most frequent and distinct patterns. We are currently exploring a follow-up analysis that will focus specifically on these less-common drusen types.

The absence of a prominent AO-TFI pattern associated with nvAMD was noted. This may stem from the complex, multilayered nature of neovascular membranes, which disrupt the optical coherence of AO-TFI and generate multiple focal planes. Our imaging protocol used a single focal adjustment per region, which may not have sufficiently captured the structural heterogeneity of nvAMD lesions. Additionally, fibrosis, hemorrhage, and exudation may obscure AO signals. While AO-TFI may detect certain features in nvAMD, these did not emerge as consistent, classifiable patterns in our current workflow. AO-SLO imaging has previously demonstrated dynamic morphologic changes in neovascular AMD, particularly before and after anti-VEGF therapy [20], yet our study did not detect salient AO-TFI patterns in this subgroup. This may reflect modality-specific limitations or the need for optimized multi-plane acquisition.

AO-TFI offers a complementary approach to other adaptive optics modalities. Compared to AO-SLO and AO-OCT, it provides larger field-of-view imaging (6.7° × 6.7°) with significantly shorter acquisition times (<10 s), making it better suited for clinical workflows [3,4,6]. AO-SLO enables exquisite resolution of the photoreceptor mosaic and has been instrumental in mapping cone density and tracking localized retinal changes, including in AMD [5,21,22]. AO-OCT, by contrast, offers depth-resolved visualization of laminar changes and subretinal pathology [17]. However, both modalities are susceptible to motion artifacts and often require complex registration and extended imaging sessions, which can limit scalability in routine practice. AO-TFI’s use of flood illumination and oblique transscleral lighting circumvents some of these challenges by capturing high-contrast *en face* reflectance images in a single shot, although it sacrifices depth resolution.

It is worth emphasizing the value of integrating AO-TFI into a broader multimodal imaging strategy. As precision medicine in ophthalmology increasingly calls for finer phenotyping, the ability to extract complementary structural information non-invasively will become critical. The unique contrast mechanism of AO-TFI—relying on tangential illumination and scattering differences—offers perspectives not accessible with OCT alone. This may be particularly valuable in the early stages of AMD, where subtle RPE remodeling or early drusen changes may not yet result in overt structural disruption on conventional cross-sectional imaging.

Moreover, AO-TFI may help bridge the translational gap between histological insights and in vivo observation [7,8,22]. Prior histological studies have shown RPE thinning, degeneration, and heterogeneous pigment accumulation in AMD, which are notoriously difficult to assess clinically [2]. High-resolution *en face* imaging, such as AO-TFI, could allow us to approximate these tissue-level changes without invasive sampling. This could ultimately lead to earlier detection of pathophysiologic changes, allowing for timely risk stratification and therapeutic intervention [23,24].

RPE integrity plays a crucial role in AMD pathophysiology. Progressive RPE dysfunction and degeneration have been implicated in both dry and neovascular AMD, driven by a combination of oxidative stress, inflammation, and lipid accumulation [25,26,27,28,29]. Our study confirms that areas corresponding to atrophy or pre-atrophy lack the typical hexagonal RPE mosaic seen in healthy retinas—a finding consistent with prior AO-TFI and AO-SLO studies [2,13]. The reflectance profiles of the patterns we observed further suggest structural alterations at the subcellular level, possibly involving RPE thinning, disruption, or dropout. While our study did not include histological correlation, previous studies have shown that high-resolution imaging can non-invasively infer structural remodeling within the outer retina and RPE [7,8].

The quantitative morphometric analysis confirmed structural progression across AMD stages. Increases in hyporeflective area, diameter, and perimeter from reticular pseudodrusen to soft drusen and pre-atrophy suggest active remodeling of subretinal structures, followed by a contraction in complete atrophy. While these metrics are mathematically related, they provide complementary insight into the spatial footprint and evolution of lesions.

From a technical standpoint, AO-TFI offers several advantages. However, limitations remain. The absence of axial resolution makes it less-suited to assessing vertical architecture or sub-RPE pathology. Furthermore, its contrast mechanisms rely on scattering properties, which may be affected by media opacities or variable tissue absorption [30]. Importantly, our study included only patients with clear media, and the image grading was performed by a single masked reader. Future work should include inter-grader variability and more robust longitudinal analysis. Additionally, improved software pipelines and possible integration with AO-SLO or AO-OCT could further enhance the clinical utility of AO-TFI.

AO-TFI represents a promising tool within the growing arsenal of high-resolution retinal imaging. By enabling fast, non-invasive visualization of fine structural changes across a broader field, it has the potential to contribute meaningfully to AMD phenotyping, particularly when used alongside complementary modalities.

## 5. Conclusions

In summary, we report the first experience of phenotyping AMD patients using an AO-TFI gray-scale background, providing a systematic description, analysis, and classification of the four most common patterns observed and their correlation with OCT findings. These initial results in AMD patients suggest the potential of AO-TFI in clinical practice, particularly as an adjunct to existing multimodal imaging to classify disease and monitor GA. However, further studies are needed to fully explore AO-TFI’s clinical potential in AMD.

## Figures and Tables

**Table 1:** Patient characteristics.

Characteristic	Summary
Number of eyes	120
Number of participants	70
Mean age (years ± SD)	78 ± 8
Sex (% female)	59%
AMD subtype	52% dry AMD (including GA), 48% nvAMD
Mean spherical equivalent (D)	−0.75 ± 1.9 (range −5.00 to +2.50)
Pupil diameter (post-dilation)	≥6 mm in all eyes
Lens status	62.5% pseudophakic, 37.5% phakic
Media clarity	Mild or no lens opacities; no vitreous haze

## Data Availability

The data supporting the findings of this study are available from the corresponding author upon reasonable request.

## Abbreviations

The following abbreviations are used in this manuscript:

AO-TFI	Adaptive optics transscleral flood illumination
AMD	Age-related macular degeneration
OCT	Optical coherence tomography
GA	Geographic atrophy
FOV	Field of view
AO	Adaptive optics
LED	Light-emitting diode
EDI	Enhanced Depth Imaging
RPE	Retinal pigment epithelium
PRs	Photoreceptors
NIR	Near-infrared
AO-SLO	Adaptive optics scanning laser ophthalmoscopy
AO-OCT	Adaptive optics optical coherence tomography
